# Successful Management of Pregnancy Complicated by Klippel-Trenaunay Syndrome Using MR Angiography-Based Evaluation

**DOI:** 10.1155/2011/723467

**Published:** 2011-10-27

**Authors:** Reiko Tanaka, Yasuyuki Fujita, Kana Ishibashi Hiasa, Yasuo Yumoto, Nobuhiro Hidaka, Kotaro Fukushima, Norio Wake

**Affiliations:** Department of Obstetrics and Gynecology, Graduate School of Medical Sciences, Kyushu University, Fukuoka 812-8582, Japan

## Abstract

Klippel-Trenaunay syndrome (KTS) is a rare congenital disease, and extensive cutaneous hemangiomas and abnormal venous vessels are characteristic. In our case, to manage her pregnancy with KTS, whole-body MRA was performed before delivery. A 29-year-old woman was referred at 28 weeks because of prominent vulvovaginal varicosities due to KTS. At 35 weeks, hypertrophy and multiple venous varicosities of her leg as well as massive vulvovaginal varicosities became prominent with a normal coagulation profile. Systematic MRAs revealed hemangiomas and varicosities in the right leg, the lower abdomen, and the pubic region, while no obvious AVM was detected around the bronchial tube and spine. We decided to deliver her baby by cesarean section at 37 weeks under general anesthesia, and a healthy baby was delivered. No blood transfusion was required. Prophylaxis against thrombosis was performed after the operation. She was discharged with her baby. Her vulvovaginal varicosities shrunk considerably one month later.

## 1. Introduction

Klippel-Trenaunay syndrome (KTS) is a rare congenital disease characterized by a triad of extensive cutaneous hemangiomas, venous varicosities, and soft tissue or bone hypertrophy affecting a leg and/or arm on one side [1]. It is often associated with massive hemorrhage, thrombosis, and pulmonary embolism due to the rupture of superficial and deep arteriovenous malformations (AVMs) during surgery. Cases of pregnancy complicated by KTS are rare, possibly because women with KTS are concerned about the cosmetic appearance of their legs. To the best of our knowledge, the English literature includes only 20 reports of pregnancy complicated by KTS, and therefore, information about the management of this condition is scarce. Herein, we present a case of pregnancy complicated by KTS that was managed by magnetic resonance angiography (MRA)-based evaluation of systemic AVMs during pregnancy and anticoagulant therapy after delivery.

## 2. Case Report

A 29-year-old woman (gravidity, 1; parity, 0) was referred to our hospital at 28 weeks of gestation because of KTS complicating her pregnancy. She had been diagnosed with KTS at the age of 3 and had not received any treatment because of the absence of symptoms. Her right leg was swollen and hypertrophic and had multiple venous varicosities. She did not have a history of thrombosis or hemorrhage. Although her pregnancy had been uncomplicated until 28 weeks of gestation, she noticed prominent right vulvovaginal varicosities and new varicosities in the right lower abdomen.

**Figure 1:**
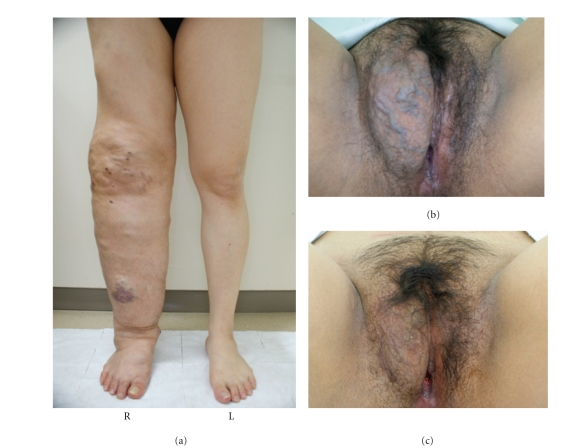
Prominent hypertrophy and multiple venous varicosities of the patient's right leg at 35 weeks of gestation (a). (R: right, L: left). The massive vulvovaginal varicosities present at 35 weeks of gestation (b) shrunk one month later (c).

On hospital admission at 35 weeks of gestation for planned delivery, physical examination showed prominent hypertrophy and multiple venous varicosities of the right leg as well as massive (15 × 10 cm) vulvovaginal varicosities (Figure 1). The circumferences of the thigh, calf, ankle, and knee were 2, 15, 11, and 10 cm, respectively, larger on the right side than on the left side. Laboratory studies revealed a normal coagulation profile (PT, 11.6 sec; APTT, 31.7 sec). The ultrasound study revealed no obvious AVMs and varicosities around the uterus. 

**Figure 2:**
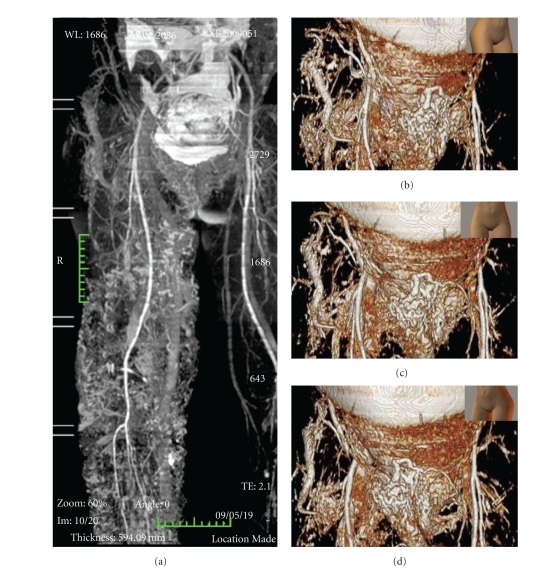
Results of MRA performed at 35 weeks of gestation. (a) Many hemangiomas and varicosities in the right leg can be seen. (b) Abnormal vessels near the anterior superior iliac spine, (c) abnormal varicosities in the right lower abdomen and upper pubic region, and (d) hemangioma near the inferior epigastric vein are also visible.

At 35 weeks of gestation, systematic MRA was performed to detect unusual AVMs. Abdominal and pelvic MRAs revealed hemangiomas and varicosities in the right leg, communications among the varicosities in the right lower abdomen, and abnormal vessels near the anterior superior iliac spine as well as unusual varicosities in the pubic region (Figures 2(a)–2(c)). In her left lower abdomen, the hemangioma associated with the inferior epigastric vein communicated with the lesions near the left anterior superior iliac spine (Figure 2(d)). MRA of the bronchial tube and spine showed no obvious AVMs. Although obvious AVMs and varicosities were not noted on the uterine surface, the possibility of spread of the venous lesions to Retzius space and the area near the rectovaginal septum was considered. 

On the basis of these findings, we decided to deliver her baby by cesarean section. For prophylaxis against thrombosis, she wore elastic stockings, and heparin calcium injection was planned after the operation. At 37 weeks of gestation, the patient underwent a cesarean section under general anesthesia by a Pfannenstiel incision to avoid rupturing any vascular anomalies at the surface of the right lower abdomen and upper pubic region. No abnormal vessels were noted around the uterine incision during the operation. A female baby weighing 2865 g was delivered; her Apgar scores were 5 and 6 at 1 and 5 min, respectively. No abnormal finding indicative of neonatal KTS was seen. The estimated blood loss including amniotic fluid was 1276 g and no blood transfusion was required.

The patient's postoperative course was unremarkable. To prevent thromboembolic disease, low-dose heparin (10,000 U/day) injection was administered for 3 postoperative days. The patient was discharged with her baby in good health. The prominent vulvovaginal varicosities shrunk considerably one month later (Figure 1(c)), although the vascular anomalies in her right leg showed no changes.

## 3. Discussion

Extensive cutaneous hemangiomas and abnormal venous vessels are characteristic features of KTS. A few reports suggest that hemorrhage during endotracheal intubation and epidural injection for anesthesia occurs because of the presence of abnormal vessels near the airway and spine, respectively [2–4]. Their presence near the birth canal or abdominal wall is also problematic during vaginal delivery and cases of disseminated intravascular coagulation with abnormal hemorrhage because such vessels have been reported [2, 5, 6]. To prevent these complications, MRI should be used to determine the existence of abnormal vessels in the pelvis, birth canal, spinal cord, bronchial tube, and brain before delivery [3, 5]. In our case, whole-body MRA before surgery revealed no obvious AVMs around the bronchial tube and spine, which helped to determine the choice of anesthesia. In addition, we could plan the incision site during cesarean section to avoid the abnormal cutaneous vascular lesions, which may explain the almost normal amount of blood loss during the operation and the absence of postsurgical complications. 

Patients with KTS have an increased risk of thromboembolic disease. Jacob et al. [7] reported that 8% of the affected patients develop pulmonary embolism or deep vein thrombosis (DVT). Further, Stein et al. [5] and Fait et al. [8] reported cases of right lower leg DVT and left calf DVT during pregnancy, respectively. Anticoagulant therapy with aspirin or heparin calcium during pregnancy could effectively prevent thromboembolic disease [5, 8–11]. Our patient was asymptomatic and was therefore not followed up for several years after diagnosis. Further, she rejected prophylactic treatment for thrombosis during pregnancy. Although our patient had a normal coagulation profile, the possibility of an abnormal coagulation profile should be considered, and anticoagulant therapy should be recommended during pregnancy.

In conclusion, some patients with KTS do not develop life-threatening complications during pregnancy. Our patient completed her pregnancy without any complications and delivered a healthy baby. To avoid the complications associated with the existence of AVMs, whole-body MRA is useful and it helps us to select the delivery mode and type of anesthesia. The MRA-based evaluation of AVMs before delivery may be a reason for the successful outcome of our patient. Pregnancy complicated by KTS may be associated with considerable risks of thromboembolic disease, which should be prevented by anticoagulant therapy. Further discussion to establish guidelines for managing pregnancy complicated by KTS is required.
